# A direct plasma assay of circulating microRNA-210 of hypoxia can identify early systemic metastasis recurrence in melanoma patients

**DOI:** 10.18632/oncotarget.3142

**Published:** 2015-02-05

**Authors:** Shigeshi Ono, Takashi Oyama, Stella Lam, Kelly Chong, Leland J. Foshag, Dave S.B. Hoon

**Affiliations:** ^1^ Department of Molecular Oncology, John Wayne Cancer Institute, Providence Saint John's Health Center, Santa Monica, CA, USA; ^2^ Division of Surgical Oncology, John Wayne Cancer Institute, Providence Saint John's Health Center, Santa Monica, CA, USA

**Keywords:** cell-free microRNA, diagnosis, plasma, metastatic melanoma recurrence, LDH

## Abstract

Circulating cell-free(cf) microRNAs (miRNAs) have been reported to exist in plasma. MicroRNA-210(miR-210) is known to play important roles in the tumor hypoxic state. We *hypothesized* that the expression levels of cf-miR-210 in plasma would predict early clinical recurrence in melanoma patients. A direct miRNA assay on plasma (RT-qPCR-DP) was developed to improve cf-miRNA assay logistics, eliminate RNA extraction, and reduce specimen amount required. RNA was extracted from formalin-fixed paraffin-embedded (FFPE) melanoma tissues (*n* = 108) and assessed by RT-qPCR. Plasma (10 μl; *n* = 264) was procured from AJCC Stage III/IV patients in phase III clinical trials. A RT-qPCR-DP was performed to detect cf-miR-210. MiR-210 was significantly higher in metastatic tumors compared to primary tumors. Cf-miR-210 was significantly higher in melanoma patients versus healthy donor controls. In serial bloods within individual patients, cf-miR-210 < 3 months prior to disease recurrence significantly increased compared to baseline levels (*p* = 0.012). ROC curve analysis demonstrated that patients with elevated cf-miR-210 were more likely to have disease recurrence. Moreover, cf-miR-210 increase significantly correlated with poorer prognosis (*p* < 0.001). Lactate dehydrogenase (LDH) level was also assessed within patients, and the AIC values for proportional hazards regression models of cf-miR-210(120.01) and LDH (122.91) demonstrated that cf-miR-210 is a better recurrence indicator. We concluded enhanced cf-miR-210 provides identification of early systemic melanoma recurrence.

## INTRODUCTION

Despite significant advances in new FDA approved drugs for malignant cutaneous melanoma, the 5-year survival of patients with American Joint Committee on Cancer (AJCC) stage IV disease is poor. AJCC stage III patients who undergo surgical resection of metastatic lymph nodes (LN) have better outcome especially when performed early after positive sentinel LN biopsy [1, 2]. To better predict systemic recurrence after the resection of regional disease, reliable blood biomarkers are highly needed. Currently the only AJCC approved blood biomarker is lactate dehydrogenase (LDH) which has been used as an indicator and progression factor in metastatic stage IV disease in melanoma for several decades. However, LDH has a limitation in accuracy and early systemic recurrence. As reported in the literature most blood cancer biomarkers predictive for systemic recurrence have not been shown to provide an accurate early time frame prior to actual clinical recurrence. We are in need of better and more efficient blood biomarkers for early systemic melanoma recurrence. This is highly important for metastatic melanomas where they are often highly aggressive in spreading and fatal. Earlier detection of systemic disease may provide an advantage for treatment such as surgery in particular recently [2], in light of new therapeutic drug regimens approved by the FDA for melanoma in the last 3 years [3, 4].

John Wayne Cancer Institute initiated prospective clinical phase III multicenter studies (the Malignant Melanoma Active Immunotherapy trials; MMAIT) in patients with AJCC stage III and IV melanoma independently to assess the efficacy of adjuvant treatment with active-specific immunotherapy. These were FDA phase III, randomized clinical trials, and patients were regularly followed up for > 5 years as defined in the clinical protocol. Patients had prospective serial bloods drawn on a defined schedule at specific intervals under Good Laboratory Practice (GLP) conditions [5, 6]. The patients were all determined to be rendered clinically disease-free before entering the trials based upon clinical assessment [5, 6]. This unique patient resource has allowed us to assess possible blood biomarkers and their utility prior to clinical evidence of recurrence. In most studies blood cancer biomarkers are assessed in patients with clinical disease present which complicates interpretation of the biomarker efficacy.

MicroRNAs (miRNAs) are small single-stranded noncoding RNA molecules that interact with their target mRNAs to inhibit translation by degrading mRNA or to block translation without degrading the mRNA by binding to complementary sequences in the 3′ untranslated regions (3′ UTR) of mRNA [7–9]. The advantage of assessing miRNAs in blood as biomarkers is that they are more stable at room temperature compared to mRNA [10], can sustain multiple freeze-thaw cycles, and survive the effects of RNase and DNase whereby mRNA degrades rapidly [11]. The remarkable stability of miRNAs in blood makes it a suitable biomarker. Expression profiles of serum/plasma miRNAs can discriminate patients with specific cancers [11–13]. While several studies have demonstrated cell-free(cf)-miRNAs potential as a biomarker in melanoma patients [14, 15], none of them has been clinically utilized or has accurately predicted recurrence in a well-defined phase III clinical trial setting patients with accurate clinical documentation and blood draws during follow-up.

We previously established a direct qPCR assay to study circulating DNA in serum from patients with breast and other cancers [16, 17]. More recently we established a RT-qPCR directly-in-serum (RT-qPCR-DS) assay for breast cancer where the RT is directly performed in serum without the need for RNA extraction [18]. Efficient and reproducible extraction of circulating cf-nucleic acids from blood has been problematic, particularly when the available amount of nucleic acids or source material is limited in quantity. The “Achilles heel” of cf-nucleic acid detection has been the extraction from body fluids, and logistical approaches of assessing miRNA have additional problems because of their small size. RT-qPCR-DS is logically simpler and more responsive in assessing cf-miRNAs than extracting RNA, as it eliminates the inevitable loss of miRNAs during the extraction step. In the current study, we applied a modified RT-qPCR directly-in-plasma assay (RT-qPCR-DP) to detect cf-miRNAs in low volume of plasma.

Hypoxia is known to be a common characteristic of the tumor microenvironment in advanced solid cancers, especially rapidly growing tumors due to inadequate blood supply in the central area of the tumor [19, 20]. MicroRNA-210 (miR-210) is one of the most significantly upregulated miRNAs in a hypoxic state, and has been reported to be an oncogenic miRNA [21–24]. It has also been shown to promote stem cell survival by targeting *CASP8AP2* [25], and to stall DNA repair by targeting *RAD52* [26], which are beneficial for cancer progression. We *hypothesized* that the expression levels of cf-miR-210 could be correlated with melanoma metastasis and be used for early identification of systemic disease recurrence.

Our studies demonstrated the utility of a direct plasma assay to detect cf-miRNA, potential of monitoring early events of clinical tumor hypoxia, and use of miR-210 in cutaneous melanoma patients to identify systemic recurrence early.

## RESULTS

### MiR-210 expression level in metastatic melanoma compared to primary tumor

**Figure 1:**
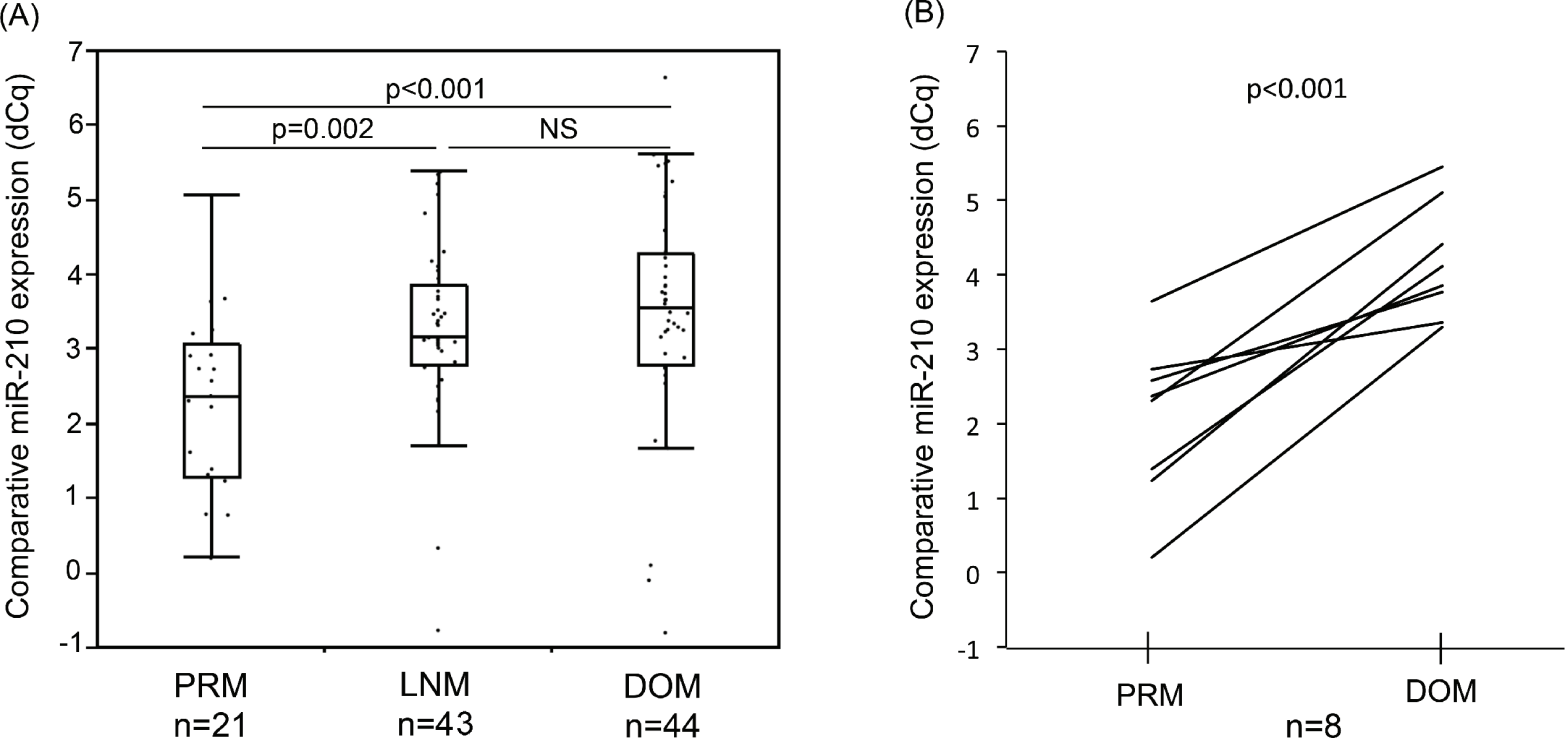
Comparison of miR-210 expression in melanoma FFPE samples **(A)** MiR-210 expression level was significantly higher in LNM or DOM melanoma samples compared to that in PRM (  *p* = 0.002, < 0.001, respectively). **(B)** In paired samples in the same patients, DOM had significantly higher miR-210 level than PRM (  *p* < 0.001).

RT-qPCR analysis of total RNA extracted from formalin-fixed paraffin-embedded (FFPE) melanomas confirmed miR-210 expression level was significantly higher in both lymph node metastasis (LNM) and distant organ metastasis (DOM) compared to primary tumors (PRM; Figure 1A; *p* = 0.002, < 0.001, respectively). Eight PRMs had paired metastases from DOM whereby we could demonstrate miR-210 level was significantly higher in DOM compared to respective PRM in all eight pairs (Figure 1B; *p* < 0.001). This demonstrated the development of the hypoxic miR-210 relevance to melanoma metastasis occurrence.

### Analytical sensitivity, specificity, and reproducibility of RT-qPCR-DP

We generated a standard curve using a serial dilution of melanoma cell line (M14) for efficiency and level of detection in the PCR assays. All plasma samples were assessed in triplicate PCR reaction. The SD of dCq values for three individual plasma samples ranged from 0.24 to 0.93 with a mean of 0.79. The samples assessed in separate RT-qPCR-DP assays produced consistent dCq values. Run 1: dCq values were (A) 5.00, (B) 7.70, and (C) 5.19. Run 2: dCq values at (A) 4.01, (B) 7.40, and (C) 5.69. Run 3: dCq values were (A) 5.32, (B) 7.62, and (C) 6.48 with the SDs of (A) 0.68, (B) 0.16 and (C) 0.65. This indicated RT-qPCR-DP could detect cf-miR-210 with high reproducibility.

A comparative assessment between with or without the Centri-Sep purification in the assay (*n* = 9; healthy donors: *n* = 5, stage IV: *n* = 4) indicated a significant increase in sensitivity (*p* < 0.001) with purification (Supplemental Figure 1).

### Cf-miR-210 expression in melanoma patients with disease present: pilot study

**Figure 2:**
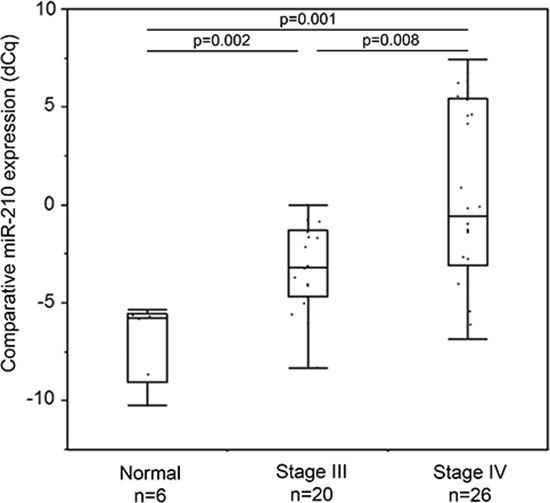
Cf-miR-210 expression in healthy donors and melanoma patients with clinical disease Cf-miR-210 level increased according to AJCC stage progression.

We then performed a pilot study to assess plasma cf-miR-210 expression in melanoma patients with different levels of metastatic disease present compared to healthy donor controls (healthy donors: *n* = 6; stage III: *n* = 20; stage IV: *n* = 26). As shown in Figure 2, cf-miR-210 could be detected by RT-qPCR-DP, and the expression level significantly increased with higher AJCC stage.

### Cf-miR-210 in disease-free patients compared to healthy donors: verification study

**Figure 3:**
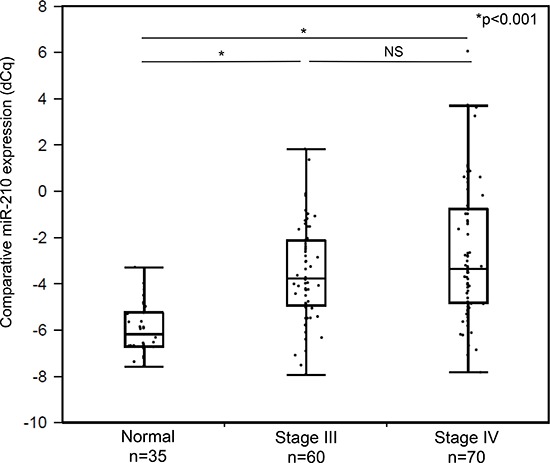
Comparison of cf-miR-210 levels in healthy donor controls and disease-free melanoma patients’ plasma samples Melanoma patients (*n* = 130) had significantly higher cf-miR-210 level than healthy donors (*n* = 35; male: *n* = 19, female: *n* = 16; *p < 0.001).

Utilizing a patient cohort A comprising of 130 AJCC Stage III (*n* = 60) and IV (*n* = 70) melanoma patients, cf-miR-210 detection in plasma was assessed by RT-qPCR-DP and compared between the disease-free melanoma patients and 35 healthy donors. The bleeding times of patients were selected based on availability. As shown in Figure 3, the cf-miR-210 level was significantly higher in the disease-free patients compared to healthy donors (*p* < 0.001).

### Cf-miR-210 expression increase before disease recurrence

Using another patient group, cohort B, comprising of AJCC stage III patients (*n* = 88) from the MMAIT-III, we compared cf-miR-210 detection in plasma taken before adjuvant treatment after being rendered disease free by surgery as the baseline, to their detection in paired serial bleeds procured from the same patient, respectively.

**Figure 4:**
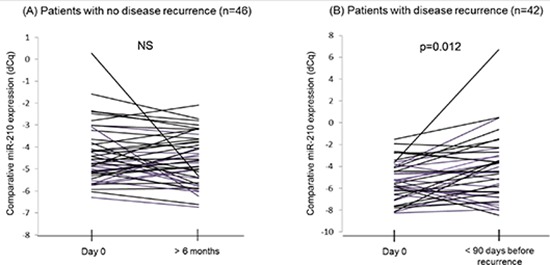
Comparison of cf-miR-210 levels in serial bleeds from the same patients **(A)** Cf-miR-210 detection in patients without recurrence did not change significantly. **(B)** Patients who recurred experienced a significant increase in cf-miR-210 detection before recurrence (  *p* = 0.012).

Cf-miR-210 expression in disease recurrent patients significantly increased prior to clinical recurrence compared to the baseline level (*p* = 0.012), however, no significant change in cf-miR-210 occurred in non-recurrent patients (Figure 4A, 4B).

No correlation was detected between the baseline cf-miR-210 expression and the length of time from surgery to the bleeding point. There was also no correlation between cf-miR-210 expression and gender or age. In addition, the type of adjuvant treatment the patient underwent (BCG + placebo or BCG + Canvaxin^®^) was not associated with patients’ outcome or cf-miR-210 expression.

### Stage III patients with elevated cf-miR-210 before systemic disease recurrence correlated to poor prognosis

**Figure 5:**
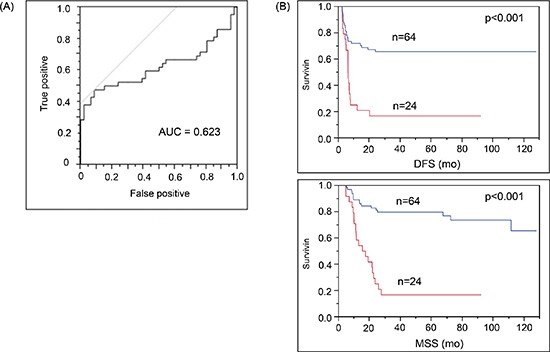
Increase in cf-miR-210 level correlated with recurrence and poor prognosis **(A)** ROC analysis: Change in cf-miR-210 was a significant predictor of recurrence (cut off: one dCq difference; Sensitivity: 47.6%, Specificity: 91.3%. AUC = 0.623; *p* = 0.015). Positive: Recurrence < 2 yrs, Negative: No recurrence > 5 yrs. **(B)** Cf-miR-210 expression was significantly related to disease outcome (DFS, MSS: *p* < 0.001). Comparison of AJCC stage III melanoma patients with either > 1 dCq increase in cf-miR-210 levels from baseline to pre-treatment bleed (*n* = 24 patients) versus patients without such an increase (*n* = 64 patients). Blue line: No increase in cf-miR-210. Red line: Increase in cf-miR-210.

In the patient cohort B, patients whose cf-miR-210 expression increased > 1 dCq from the baseline to their paired plasma were significantly more likely to recur. Receiver-operating characteristics (ROC) curve analysis was performed, and AUC was 0.623 (*p* = 0.015; Figure 5A). The Stage III patients with enhanced cf-miR-210 expression also experienced significantly poorer prognosis compared to patients whose cf-miR-210 expression did not significantly change. The differences in disease-free survival (DFS) and melanoma-specific survival (MSS) were highly significant (DFS: *p* < 0.001; MSS: *p* < 0.001; Figure 5B). An increase in cf-miR-210 expression in plasma was not associated with the adjuvant treatment type the patient received.

### Cf-miR-210 compared to lactate dehydrogenase (LDH)

**Figure 6:**
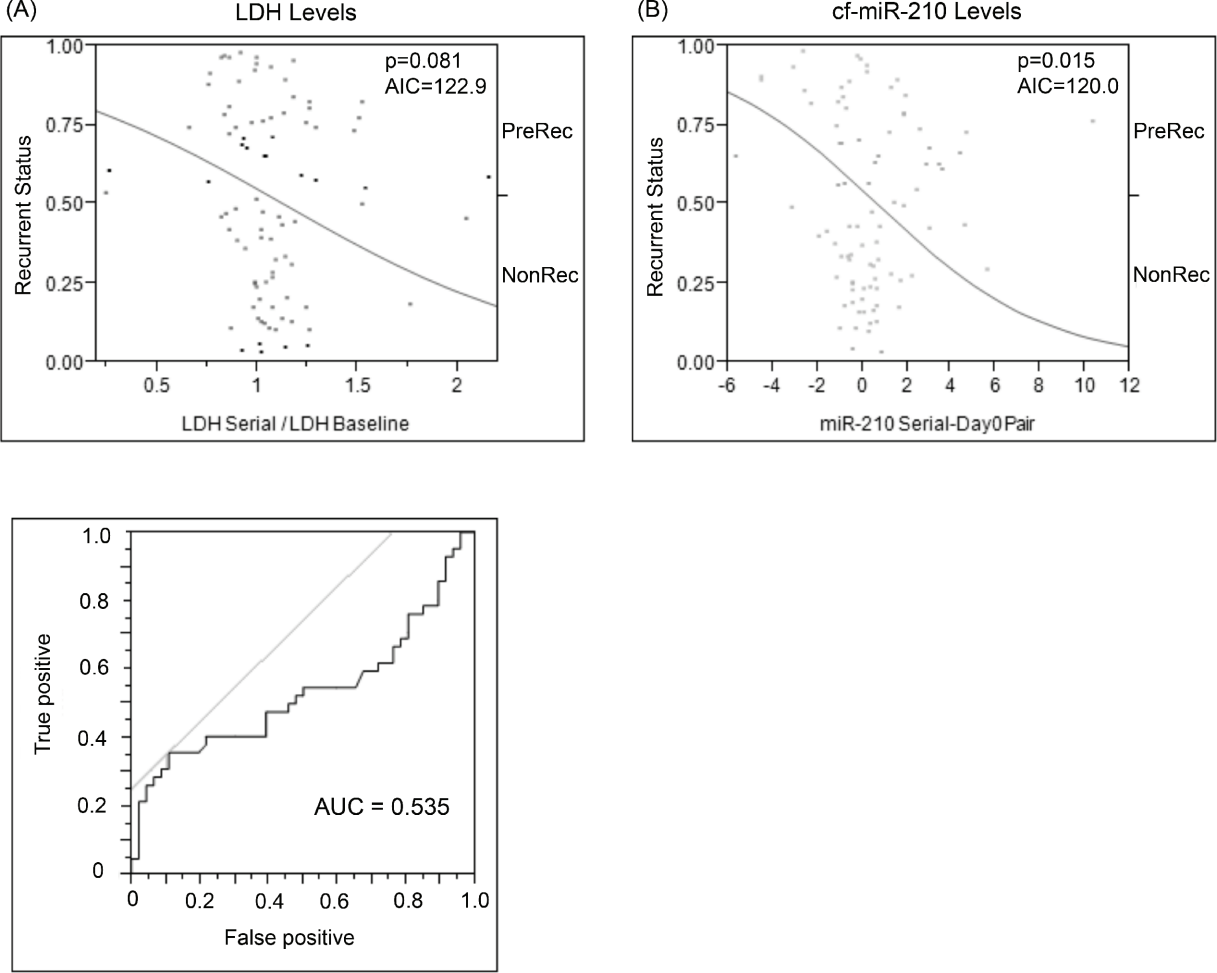
Comparison of change in baseline LDH vs change in cf-miR-210 dCq **(A)** ROC analysis was performed to determine the cutoff for LDH changes (LDH Cutoff: 1.175-fold change from baseline; AUC = 0.535). Change in LDH level did not predict recurrence (*  p* = 0.810, AIU = 122.91) (A), while change in cf-miR-210 was a significant predictor of recurrence **(B)** (  *p* = 0.15, AIU = 120.01). Positive: Recurrence < 2 yrs, Negative: No recurrence > 5 yrs.

LDH levels of the recurrent patients in blood were also analyzed. It was from either the same day or < 30 days of the plasma for which cf-miR-210 was assessed. Although elevated LDH showed a trend for predicting disease recurrence, it was not significant (*p* = 0.081; Figure 6A). However, an increase in cf-miR-210 was a significant predictor of disease recurrence (*p* = 0.015; Figure 6B). Akaike information criterion (AIC) values for proportional hazards regression models of cf-miR-210 was lower than that of LDH (AIC = 120.01, 122.91, respectively), which demonstrated cf-miR-210 was the better indicator of disease recurrence. Combination of cf-miR-210 and LDH did not significantly improve performance for prediction of melanoma recurrence (AIC = 120.03).

## DISCUSSION

There are several tissue-based, blood-based and *in vitro* functional studies of miRNAs in melanoma [14, 27–31], however, the role of hypoxic miR-210 in melanoma remains unclear. In addition, there has been no significant report about cf-miR-210 in melanoma related to monitoring disease outcome.

MiR-210 is the most eminent hypoxia-inducible miRNA that is over-expressed in several cancers [24]. Rapidly proliferating tumors may outgrow their blood supply giving rise to hypoxia within the tumor that causes over-expression of miR-210 for angiogenesis [19, 20, 32]. In addition, several reports have indicated that miR-210 is correlated with a poor prognosis in various cancers [24]. In this study, we described the efficacy of measuring cf-miR-210 in plasma as a prognostic biomarker for early systemic disease recurrence for the first time.

We confirmed that the level of miR-210 in melanomas tissues was significantly higher in metastases compared to PRMs. There was also a significant difference between the paired-PRM and DOM melanomas within patients. These results implied miR-210 was elevated during systemic disease progression. It has been also reported that hypoxia is related to disease progression through vascular endothelial growth factor (VEGF) induction and accompanied angiogenesis and metastasis [33, 34]. Metastasis develops through multiple events including angiogenesis [35], and miR-210 is also well-known to induce angiogenesis after exposed to hypoxia [32]. Our finding could demonstrate hypoxia-inducible miR-210 has an important role during melanoma disease progression.

Cf-miR-210 level was elevated in the plasma of melanoma patients compared to healthy donor control plasma. These results suggested cf-miR-210 could be an indicator for systemic metastasis. In cohort A, there was no significant difference in cf-miR-210 expression between stage III and IV, this is likely because the patients had no presence of clinical disease at the time of blood draw. In addition, we demonstrated elevated cf-miR-210 was seen in the patients who subsequently developed systemic recurrence prior to their clinical diagnosis, and therefore could predict recurrence earlier.

In cohort B, we utilized the blood taken after the curative resection of melanoma as the baseline. Tumor derived cf-miRNA levels have been demonstrated to drop significantly seven days after surgery [36]. Cf-miRNA expression level in our pretreatment plasma was not likely affected by the surgery itself, gender and age, or melanoma-derived from clinically evident disease because the bleeding point was at least 13 days after the definitive surgery. This implies that cf-miR-210 level may be higher in melanoma patients even after the curative resections compared to healthy donor control, possibly from clinically occult tumors. Considering this finding, it is quite important to follow the expression level serially to detect systemic progression of the disease. One of the major problems after surgical resection of melanoma tumors to render the patient clinically disease-free is the follow-up and identification of early systemic disease recurrence. With multiple FDA approved drugs, effective combinations of drugs, as well as many new phase II/III drugs in the pipeline, melanoma patients have many more opportunities of having extended survival than several years ago. Therefore, the earlier detection of disease recurrence is even more important; such that treatment can be assigned appropriately to result in prolong survival.

LDH levels were also analyzed in plasma taken on either the same day, or < 30 days for which cf-miR-210 was assessed. LDH is currently the only approved biomarker routinely used in assessing for and level of metastasis in melanoma patients. Elevated level of LDH in serum is an independent and highly significant negative predictor of survival outcome among stage IV patients [1] even though it has been a second determinant of distant metastasis staging in melanoma [37, 38]. In addition, it is rarely a singular indicator of disease recurrence and survival prediction for any other melanoma patients except for AJCC Stage IV patients [1]. In this study, cf-miR-210 was the only predictor of disease recurrence for Stage III melanoma patients, raising the possibility that cf-miR-210 could potentially be a novel biomarker for predicting disease recurrence or prognosis of these melanoma patients.

In conclusion, increasing cf-miR-210 in advanced melanoma patients is a prognostic biomarker of risk of recurrence and for disease outcome that may allow us to monitor patients better and begin treatment earlier. The RT-qPCR-DP is a sufficient assay to detect different levels of miRNA in small volumes of blood. The ability to detect and quantify cf-miR-210 may ultimately assist in clinical decision making such that it can be performed at multiple times to assess disease response to various treatments and aid in the earlier detection of systemic recurrence.

## MATERIALS AND METHODS

### FFPE tissue analysis

All FFPE tissue samples assessed were obtained from 108 patients at Saint John's Health Center (SJHC) between 1995 and 2010 (Supplemental Table 1). Tissue samples were fixed and embedded using the SJHC department of pathology's the Tissue-Tek^®^ VIP^®^ 6 Processing for Histology protocol. The samples consisted of PRM (*n* = 21), LNM (*n* = 43), and DOM (*n* = 44). DOM sites were lung (*n* = 16), bowel (*n* = 12), liver (*n* = 6), distant skin (*n* = 3), brain (*n* = 2) and others (*n* = 5). All samples and clinical information were obtained in accordance with the SJHC/John Wayne Cancer Institutional (JWCI) Review Board (IRB) approval.

### Patients in the clinical trial and blood collection

In the MMAIT, patients after the complete resection of AJCC stage III/IV melanoma were randomly assigned to the two treatment arms: BCG plus a Canvaxin^®^ melanoma vaccine or BCG plus a placebo [5, 6]. The clinical trials were registered as FDA phase III trials, with two trials for AJCC stage III and IV cutaneous melanoma between 1998 and 2005 [5, 6]. All the clinical data for patients were prospectively collected after informed consent and under FDA guidelines. BCG with or without vaccine was given as an immune adjuvant as previously described [5]. The blood specimens were collected before adjuvant treatment and at several serial bleed defined points, under GLP conditions and during adjuvant treatment. Finally, the trial showed no clinical difference between the two randomized treatment arms.

Initially three different patients’ plasma samples from AJCC stage III were used to assess whether RT-qPCR-DP can detect cf-miRNA in plasma and show reproducibility. For the pilot study, plasma samples were obtained from six healthy donors and AJCC stage III (*n* = 20) and IV (*n* = 26) melanoma patients with disease present. For the verification study, plasma samples were obtained from healthy donors (*n* = 35), and 218 clinically well-annotated patients were selected, which comprised two patient cohorts (A: *n* = 130; B: *n* = 88), from the MMAIT. Patient cohort A contained 130 AJCC stage III (*n* = 60) and IV (*n* = 70) disease-free patients with specific bleed points (Supplemental Table 2A, 2B). The cohort was utilized for comparing cf-miR-210 expression level between disease-free melanoma patients and healthy donors. Cohort B contained 88 stage III patients, separated by those that either recurred < 2 years after surgery (*n* = 46) or that did not recur for > 5 years after surgery (*n* = 42; Table 1). In cohort B, paired serial bleeds from the same patients were assessed beginning prior to adjuvant treatment, and either < 90 days prior to recurrence for recurrent patients or > 6 months after adjuvant treatment started for non-recurrent patients, to determine if cf-miR-210 detection changed prior to the recurrence. The pretreatment bleed points ranged from 13 to 95 days after the surgery.

**Table 1:** Patient characteristics

Clinicopathological Factor	# Patients (%)
**Treatment Arm**	
**Canvaxin^®^**	**36 (41%)**
Recurrent (within 2 years)	16 (44%)
Nonrecurrent (> 5 years)	20 (56%)
**Placebo**	**52 (59%)**
Recurrent (within 2 years)	26 (50%)
Nonrecurrent (> 5 years)	26 (50%)
**Gender**	
Male	56 (64%)
Female	32 (36%)
**Age (Mean ± Std)**	49.3 ± 14.1
**Palpable LN Status**	
Palpable	43 (49%)
Non-palpable	45 (51%)
**LN Positive**	
1 positive	45 (51%)
2–3 positive	24 (27%)
4+ positive	19 (22%)
**Primary Tumor Ulceration**	
Yes	32 (36%)
No	31 (35%)
Unknown	25 (28%)
**Maximum LDH (Mean ± Std)**	318 ± 202
**Primary Tumor Breslow Thickness**	
≤ 1.00 mm	16 (18%)
1.01–2.00 mm	18 (20%)
2.01–4.00 mm	22 (25%)
> 4.00 mm	24 (27%)
Unknown	8 (9%)
**Primary Tumor Site**	
Head/Neck	9 (10%)
Trunk	39 (44%)
Extremity	33 (38%)
Other	3 (3%)
Unknown	4 (5%)

This study was performed in concordance with the Reporting Recommendations for Tumor Marker Prognostic Studies (REMARK) of the National Cancer Institute [39].

### Blood specimens

The clinical trial and companion blood studies were approved by the IRB at SJHC/JWCI and all other participating centers. All blood specimens were taken together under a specific standard operating procedure (SOP) approved by the MMAIT cohort for all sites and monitored accordingly for quality control. Blood processing was performed under SOP in GLP conditions predetermined prior to the start of the clinical trial. Peripheral blood was collected in 4.5 ml blue-top tubes containing anticoagulant sodium citrate. Blood samples were centrifuged (1,300 × g, 10 min.) to separate plasma, plasma was carefully collected, and then immediately processed through a 13-mm filter to remove potential cell contamination and blood related debris. Plasma was then aliquoted and cryopreserved in liquid nitrogen under GLP conditions until used for this study.

### RNA extraction from FFPE tissue samples

For RNA extraction, 10 sections at 10 μm thick were cut from each FFPE tissue block. Deparaffinized tissues were digested with Proteinase K (Applied Biosystems) at 50°C for 3 hrs as previously described [40]. Samples were homogenized and lysed in RNA-Solv Reagent (Omega Bio-Tek) followed by chloroform to denature proteins. Overnight precipitation was performed with isopropanol in addition to Pellet Paint NF (Novagen) as a carrier to pellet the RNA. Total RNA yield was determined and assessed for purity by using ultraviolet spectrophotometry and Quant-iT RiboGreen RNA assay kit (Life Technologies).

### Reverse transcriptional quantitative PCR (RT-qPCR)

Reverse-transcription of 250 ng of total RNA extracted from FFPE samples was performed using qScript cDNA Synthesis Kit (Quanta Biosciences). The transcribed cDNA was diluted 10-fold with nuclease-free water after the mixture was incubated at 37°C for 2 hrs.

Each qPCR (PerfeCTa^®^ microRNA Assay; Quanta Biosciences) contained 4.6 μl diluted cDNA, 5 μl of 2x PerfeCTa SYBR Green SuperMix (Quanta Bioscience), 0.2 μl miR-specific primer, and 0.2 μl PCR Universal primer. Samples were assessed in triplicates at 95°C for 2 min: 40 cycles at 95°C for 5 sec and 60°C for 30 sec. A CFX96 Touch™ Real-Time PCR Detection System (BIO-RAD) was used for qPCR with melting-curve analysis. Each sample was assessed in triplicates with positive and negative cell lines, plasma controls and individual reagent controls to be used as inter-plate correction. The expression level of miRNA was recorded as dCq as follows: dCq = mean Cq values (1 ng of RNA from M14) − mean Cq values (each sample).

### RT-qPCR-DP

RT-qPCR-DP was performed with 10 μl of plasma. Each plasma sample was mixed with 10 μl of 2.5% Tween as previously described [18] to deactivate and solubilize proteins that would affect the RT-qPCR results. In addition, it dissolves exosomes and lipid bound miRNA in the plasma. In the RT step, 20 μl of RT reagent mixture containing 5X first strand buffer (PROMEGA), 10mmol/L deoxynucleoside-5′-triphoshate, miRNA-specific RT primer, RNasin, Moloney murine leukemia virus reverse transcriptase, and nuclease-free water was added to each plasma sample followed by a 2 hrs incubation at 37°C with a final 5 min enzyme inactivation at 95°C. The transcribed cDNA was centrifuged at 9,000 g for 5 min to eliminate the protein precipitate, and then the supernatant was purified using Centri-Sep 8 well strips kit (Princeton Separations) according to the manufacturer's instruction. The Centri-Sep removes lipids, inhibitor proteins, RT and nucleic acid impurities, and aids in purification. It also eliminates free and labeled dNTPs from DNA or RNA. DNase treatment was not necessary for this assay as previously described [18].

Each qPCR reaction contained 4.5 μl of cDNA, 5 μl of PerfeCTa SYBR FastMix for iQ (Quanta Bioscience), 0.2 μl of miRNA-specific forward primer, 0.2 μl of universal reverse primer, and 0.1 μl of nuclease-free water. Samples were assessed in triplicates in 40 cycles at 95°C for 10 sec and of 42°C for 30 sec. Positive and negative control, in addition to reagent controls were implemented and used as inter-plate correction. Standard curves were generated by using five serially diluted (0.1–10 ng) M14 Cq values. The comparative quantification of miRNA expression level was recorded as the dCq as described above.

Due to the small nature of these miRNAs, conventional RT and RT-qPCR cannot be used to accurately detect miRNAs. A short unique RT-Specific primer is designed to target the 3′ end of miRNAs while a universal tag is designed to the 5′ end of the RT-Specific primer. The universal PCR reverse primer complements the universal tag that was added to the RT step. PCR universal reverse primer sequence: 5′-CATGATCAGCTGGGCCA-3′. The miR-210 primer sequence was: 5′-CATGATCAGCTGGGCCAAGATCAGCCG-3′ (rt-specific); 5′-CTGTGCGTGT-3′ (forward).

### Biostatistical analysis

The expression levels of miRNAs were compared by using Wilcoxon Rank Sum test for individual bleed points and using the paired *t*-test for comparing serial bleeds within patients. Pearson product-moment correlation coefficient was used to analyze correlations between two groups. ROC curve was established to evaluate the diagnostic value of plasma miRNAs for the differentiation between melanoma recurrent patients and non-recurrent patients. The Fisher's exact test was used in the analysis of contingency tables. Univariate survival analysis was performed by applying the log-rank test to cf-miRNA expression levels and standard melanoma prognostic factors. A *p*-value of < 0.05 was considered statistically significant. All analysis was performed with JMP 10 software (SAS Institute).

## SUPPLEMENTAL FIGURE AND TABLES



## Abbreviation

AIC: Akaike information criterion
AJCC: American Joint Committee on Cancer
AUC: area under the curve
BCG: Bacillus Calmette-Guerin
cf: cell-free
DFS: disease-free survival
FFPE: Formalin-fixed paraffin-embedded
IRB: Institutional review board
LDH: lactate dehydrogenase
miRNA: microRNA
MMAIT: Malignant Melanoma Active Immunotherapy Trial
MSS: melanoma specific survival
qPCR: quantitative polymerase chain reaction
REMARK: Reporting Recommendations for Tumor Marker Prognostic Studies
ROC: receiver-operating characteristics
RT: reverse transcription
RT-qPCR-DP: RT-qPCR directly in plasma assay
SOP: standard operating procedure
3′UTR: 3′ untranslated regions
