# Supplementary material for: A direct plasma assay of circulating microRNA-210 of hypoxia can identify early systemic metastasis recurrence in melanoma patients
Source: Oncotarget. 2015 Feb 5;6(9):7053–64. doi: 10.18632/oncotarget.3142 (PMC4466669; doi:10.18632/oncotarget.3142)
Supplement: Supplementary file 1 [file oncotarget-06-7053-s001.pdf]

SUPPLEMENTAL FIGURE AND TABLES

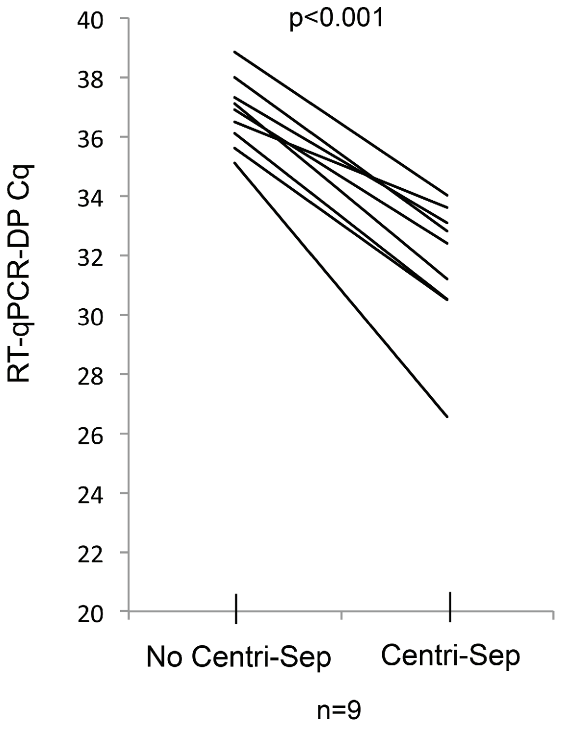

Supplemental Figure 1: Comparison of RT-qPCR-DP Cq with and without Centri-Sep kit purification. Purification with Centri-Sep kit significantly improved RT-qPCT-DP sensitivity (n = 9; healthy donors: n = 5; stage IV: n = 4; $p < 0.001$).

Supplemental Table 1: Patient characteristics of FFPE samples assessed

Clinicopathological Factor	# Patients (%)
**Tissue Group**	
PRM	21 (19%)
LNM	43 (40%)
DOM	44 (41%)
**Gender**	
Male	68 (63%)
Female	40 (37%)
**Age (Mean ± Std)**	57.8 ± 12.8
**Primary Tumor Site**	
Head/Neck	27 (25%)
Trunk	27 (25%)
Extremity	35 (32%)
Mucosal	2 (2%)
Unknown	17 (16%)
**Primary Tumor Breslow Thickness**	
≤1.00 mm	18 (17%)
1.01–2.00 mm	17 (16%)
2.01–4.00 mm	17 (16%)
>4.00 mm	24 (22%)
Unknown	32 (30%)
**Primary Tumor Ulceration**	
Yes	30 (28%)
No	36 (33%)
Unknown	42 (39%)

PRM: primary tumor; LNM: lymph node metastasis; DOM: distant organ metastasis.

Supplemental Table 2A: Patients characteristics

Stage III Patients (*n* = 60)	# Patients (%)
**Treatment Arm**	
Canvaxin®	31 (52%)
Placebo	29 (48%)
**Gender**	
Male	46 (77%)
Female	14 (23%)
**Age (Mean ± Std)**	55.6 ± 14.2
**Palpable Node Status**	
Palpable	40 (67%)
Non-palpable	20 (33%)
**LN Positive**	
1 positive	21 (35%)
2–3 positive	23 (38%)
4+ positive	16 (27%)
**Primary Tumor Ulceration**	
Yes	22 (37%)
No	17 (28%)
Unknown	21 (35%)
**Maximum LDH (Mean ± Std)**	375 ± 504
**Primary Tumor Breslow Thickness**	
≤1.00 mm	10 (17%)
1.01–2.00 mm	13 (22%)
2.01–4.00 mm	13 (22%)
>4.00 mm	14 (23%)
Unknown	10 (17%)
**Primary Tumor Site**	
Head/Neck	7 (12%)
Trunk	24 (40%)
Extremity	21 (35%)
Other	1 (2%)
Unknown	7 (12%)

Supplemental Table 2B: Patients characteristics

Stage IV Patients (n = 70)	# Patients (%)
**Treatment Arm**	
Canvaxin®	33 (47%)
Placebo	37 (53%)
**Gender**	
Male	42 (60%)
Female	28 (40%)
**Age (Mean ± Std)**	52.9 ± 12.7
**Number of DOM**	
1	39 (56%)
2–3	30 (43%)
4	1 (1%)
**Maximum LDH (Mean ± Std)**	326 ± 201
**Primary Tumor Breslow Thickness**	
≤1.00 mm	11 (16%)
1.01–2.00 mm	14 (20%)
2.01–4.00 mm	13 (19%)
>4.00 mm	11 (16%)
Unknown	21 (30%)
**Primary Tumor Site**	
Head/Neck	7 (10%)
Trunk	24 (34%)
Extremity	22 (31%)
Other	2 (3%)
Unknown	15 (21%)

DOM: distant organ metastasis.
